# Superfast Set, Strong and Less Degradable Mineral Trioxide Aggregate Cement

**DOI:** 10.1155/2017/3019136

**Published:** 2017-10-19

**Authors:** Abdullah Alqedairi, Carlos A. Muñoz-Viveros, Eugene A. Pantera, Marc Campillo-Funollet, Hussam Alfawaz, Ensanya Ali Abou Neel, Tariq S. Abuhaimed

**Affiliations:** ^1^Division of Endodontics, Department of Restorative Dental Sciences, King Saud University, Riyadh, Saudi Arabia; ^2^Department of Restorative Dentistry, SUNY at Buffalo, Buffalo, NY, USA; ^3^Division of Endodontics, Department of Periodontics and Endodontics, SUNY at Buffalo, Buffalo, NY, USA; ^4^Division of Biomaterials, Operative Dentistry Department, King Abdualziz University, Jeddah, Saudi Arabia; ^5^Biomaterials Department, Faculty of Dentistry, Tanta University, Tanta, Egypt; ^6^Division of Biomaterials and Tissue Engineering, UCL Eastman Dental Institute, 256 Gray's Inn Road, London WC1X 8LD, UK

## Abstract

**Purpose:**

Despite the good sealing ability and biocompatibility of mineral trioxide aggregate (MTA), its slow setting, high degradation, and weakness limit its use in surgical endodontics and high stress-bearing areas. This study aimed to develop two new liquids to control these drawbacks. They were prepared from calcium chloride, fumed silica, and hydroxyapatite or calcium phosphate and coded “H” and “P,” respectively.

**Methods:**

Portland cement, Grey ProRoot® MTA, and white ProRoot MTA were mixed with distilled water (control) or liquid “H” or “P.” The pH, setting time, degradation rate, leachant/precipitate' composition, compressive strength, and morphology were assessed.

**Results:**

Both liquids maintained MTA's high alkalinity and reduced the setting time by 1-2 orders of magnitude. Both liquids, H in particular, significantly reduced the degradation rate of Grey ProRoot and White ProRoot MTA®. Calcite has been identified as the main phase of the leachant or precipitate formed during the cement's degradation. Calcium hydroxide or hydroxyapatite was also identified with Grey ProRoot MTA mixed with H liquid. These liquids also significantly increased the compressive strength with no statistical differences between them; this was associated with the production of dense, consolidated structures.

**Conclusions:**

The modified MTA could be used in surgical endodontics and high stress-bearing areas.

## 1. Introduction

Portland cement (PC) is a mixture of di- and tricalcium silicate, tricalcium aluminate, and tetracalcium aluminoferrite [1]. Mineral trioxide aggregate (MTA) is a type I PC that contains 50–75 wt% calcium oxide (CaO) and 15–25 wt% silicon dioxide (SiO_2_) [2, 3]. MTA was developed by Lee and coworkers 1993 [4] and FDA approved as vital pulp capping material in 1997 [5] and as a perforation repair material and apical plug during apexification in 1998 [6]. Furthermore, the FDA supported the safety and effectiveness of MTA as a root-end filling material [7]. MTA was initially available in the market as a grey-colored ProRoot MTA (GMTA). Due to aesthetic concerns with GMTA, a white ProRoot MTA (WMTA) formulation was introduced in 2002 [8]. Unlike PC, both GMTA and WMTA contain 20% bismuth oxide as radiopacifier [9] and calcium sulfate (CaSO_4_, 5 wt%) to regulate their setting time [10, 11].

Despite its good sealing ability [12], osteoconductivity [13], and high biocompatibility [14], MTA has some disadvantages such as long setting time, high degradation, and low compressive strength [15]. Due to its long setting time and the need for water to complete the hydration (setting) reaction, a wet cotton pellet is usually placed next to MTA; the placement of the definitive filling material will therefore be delayed [16]. Furthermore, in surgical endodontics, when used as root-end filling materials, MTA can be easily washed out during the irrigation step [17]. Due to its high degradation and weakness, MTA is usually used under other materials and cannot be used in high stress-bearing areas.

This study aimed to investigate the efficacy of two novel liquids designed to decrease the setting time, reduce the degradation, and improve the strength of MTA. These liquids were prepared as aqueous suspensions of calcium chloride_,_ fumed silica, and hydroxyapatite or calcium phosphate and coded as “H” and “P” on the pH, setting time, degradation rate, compressive strength, and internal morphology of PC, GMTA, and WMTA. The null hypothesis was “the two novel liquids do not affect the properties of the cement mentioned above.”

## 2. Materials and Methods

For this study, three cement types were used; they are presented in Table 1. These cement types were provided as powder that mixed with distilled water. Two experimental liquids were used instead of “only” distilled water. The composition of these liquids and the material codes are presented in Table 1. These cement types were prepared according to the manufacturer's instructions at powder : liquid ratio of 3 : 1 (except for the pH study). The weights of chemicals used for preparing both experimental liquids were considered from the powder component of MTA to maintain the powder : liquid ratio of 3 : 1. After mixing, the cement was then loaded into a split stainless steel mold of different dimension according to the test. The mold was supported by a glass slab at the top and bottom to produce samples with flat and smooth surfaces. Samples mixed with distilled water were used as controls.

### 2.1. pH Analysis

For this study, five suspensions for each cement type were prepared by mixing 1 g of powder with 30 ml of each liquid. The suspension was continuously agitated using a stirrer to prevent its hardening. This method was used to detect the pH changes during setting of each cement type. The pH reading was recorded immediately after mixing and then every 10 s for 30 min using a microprocessor-based pH meter (HI221-pH meter; Hanna Instruments Inc., Woonsocket, USA).

### 2.2. Final Setting Time

A modified ANSI/ADA Specification Number 9 [18] method was used to measure the final setting time of tested materials. A split mold (10 mm diameter and 5 mm thickness) was used to prepare samples (*n* = 5 for each cement type). During testing, samples were incubated in a custom made acrylic chamber of 95% relative humidity and 37 ± 2°C. The final setting time of all samples were measured inside the chamber using a Gillmore needle (Humboldt Mfg. Co., Norridge, USA) with a standard weight of 453.6 ± 0.5 g and 1 ± 0.1 mm tip diameter. The time between the start of mixing until the indenter needle leaves a barely predictable mark on the cement's surface was considered as the setting time.

### 2.3. Degradation Study

A modified ANSI/ADA Specification Number 9 [18] method was used to determine the degradation of tested materials. Samples (*n* = 6 for each cement), prepared as described above, of 10 mm diameter and 1 mm thickness were used for this study. Samples were incubated at 37°C and 100% relative humidity until complete setting as determined from the previous test. Samples were then submerged in a glass bottle using a stainless steel ligature wire, passing through a central hole made in each sample before setting. The whole assembly including the sample, wire, and the bottle was weighed. The initial weights of both wire and bottle were subtracted from the total weight of the whole assembly in order to obtain the initial weight of each sample. Then 50 ml of HPLC grade water (Avantor Performance Materials, Inc., USA) was added in each bottle; the whole assembly was then kept in an incubator at 37°C. At different time intervals (1, 3, 7, 14, and 28 days), the bottles were removed from the incubator; the specimens were then flushed with a small amount of HPLC grade water, which was recollected in the same bottle. The water in these bottles was then evaporated at 90°C. The bottles were then dried at 110°C overnight. After cooling to room temperature in a desiccator containing silica gel, the bottle and its contents were weighed using an analytical balance (AG204, Mettler-Toledo, Switzerland) with a precision of ±0.2 mg. The cycle of heating to 110°C, cooling over a desiccant, and reweighing was repeated until the weight loss of each bottle was not more than 0.5 mg in any 24-hour period. The weight loss was calculated from the difference between the final and the initial weight of the weighing bottle. The data was plotted as cumulative weight loss (%) against time. From the plot, the degradation rate was calculated.

### 2.4. X-Ray Diffraction Analysis

After the degradation study, the precipitates, collected from the surface of each sample during the washing step as well as those left in the bottle of GMTA-W and GMTA-H samples, were analyzed using X-ray diffraction analysis (XRD, Ultima IV, Rigaku, Japan) at 2*θ* of 20–70° and at a rate of 1°/min. A Cu-*α* X-ray source with an acceleration voltage of 40 kV and an electron beam current of 44 mA was used. Search-match software was used to identify phases utilizing the International Center for Diffraction Data (ICDD) database, 2011-2012. In order to obtain enough precipitate to perform the X-ray analysis, the precipitate collected from several specimens of the same group was merged into one sample. The precipitates detected on the surface of specimens and seen as crystals were easily detached from the surface using a small brush. Those left in bottles used for the degradation study were collected as described above.

### 2.5. Compressive Strength

A modified ANSI/ADA Specification Number 8 [19] method was used to measure the compressive strength of tested materials. Samples (*n* = 10 for each cement), prepared as described above, of 10 mm diameter and 6 mm thickness were used for this study. After complete setting, samples were smoothened with fine-grain sandpapers (600-grit) and kept in distilled water at 37°C for 24 h. Before testing, samples dimensions were measured using a micrometer. The compressive strength measurement was conducted using a universal testing machine (858 Mini Bionix II, MTS Systems Corporation, USA) at a cross head speed of 1 mm/min. During testing, samples were kept wet using a wet blotting paper. The compressive strength (*σ*) in “MPa” was calculated using the following equation:

(1) $$\begin{array}{c} \sigma =\frac{4P}{\Pi d^{2}}, \end{array}$$

where “*P*” is the maximum applied load in “Newton” and “*d*” is the diameter in “mm.”

### 2.6. Scanning Electron Microscopy

The internal morphology of a freshly fractured surface of set specimens was analyzed using scanning electron microscopy (SEM-SU4000, Hitachi, Japan) after being mounted on aluminum stubs and coated with carbon.

### 2.7. Statistical Analysis

Statistical analysis was carried out using Student's *t*-test at a 5% significance via IBM SPSS20 (Chicago, USA). Student's *t*-test was adjusted using Bonferroni's method when there was an indication of variance nonhomogeneity (Levene's test).

## 3. Results and Discussion

Although MTA has proved to be a material of choice for many clinical applications in dentistry, its long setting time, weakness, and high degradation are the major disadvantages that may jeopardize its integrity. This study aimed to investigate the efficacy of two new liquids on the pH, setting time, degradation rate, compressive strength, and internal morphology of PC, GMTA, and WMTA. These liquids are aqueous suspensions of calcium chloride, fumed silica, and hydroxyapatite or calcium phosphate. Calcium chloride with its unique cation-anion (Ca^+2^/Cl^−^) combination is one of the best accelerators for the hydration of calcium silicate, the main component of MTA [20]. The fumed silica is expected to react with calcium hydroxide (one of the hydration reaction products) and form more of the calcium silicate hydrate that acts as a binder [21, 22]. This binder will eventually hold all reaction products and prevent their leaching (i.e., reduces degradation). Hydroxyapatite or calcium phosphates will help in the hardening of the formed cement and hence in the strength development of the cement [23].

### 3.1. pH Analysis

**Figure 1:**
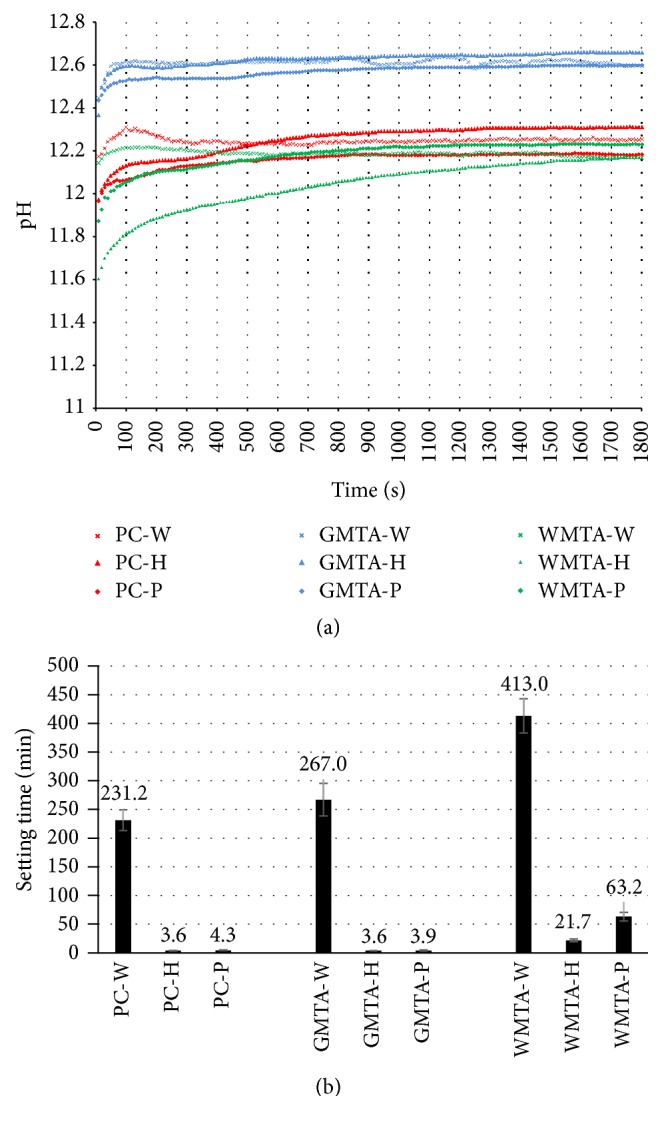
Mean pH values against time (s) (a) and final setting time (min) (b) for PC, GMTA, and WMTA cement groups.

To detect the effect of two liquids used in this study on the pH of the cement during its setting, cement suspensions were used. The suspension was continuously agitated using a stirrer to prevent its hardening during pH measurements. The mean pH values at various time intervals for all tested groups are presented in Figure 1(a). The starting pH varies from 11.60 to 12.4 according to the type of cement; a gradual increase in pH was then observed with time. After 30 min, the pH value reaches 12.2–12.7 according to the type of cement. Generally, GMTA cement group shows higher pH value than the other two cement groups. This finding indicated that all cement types are alkaline (i.e., pH above 11); this high pH could be attributed to the formation of calcium hydroxide during hydration process [24].

Using liquid “H” or “P” instead of water has no significant effect on immediate and 30 min pH except WMTA-H that has significantly (*p* < 0.05) lower immediate pH value than WMTA-W. Regardless of this reduction, the pH of WMTA-H cement is still alkaline. This finding indicated that the use of both experimental liquids has no adverse effect on the alkalinity of the tested cement. This high pH is responsible for the high biocompatibility and antibacterial action of MTA [15]. According to this finding, there is no reason to reject the null hypothesis for pH.

Comparing the effect of both liquids “H” and “P,” there was no significant difference in the immediate or 30 min pH values of cement mixed with H or P liquid except that PC-H has significantly (*p* < 0.05) higher 30 min pH than PC-P (Table 1).

### 3.2. Final Setting Time

As shown in Figure 1(b), WMTA-W cement has significantly longer (*p* < 0.05) final setting time than PC-W and GMTA-W cement. Generally, the final setting time for PC, GMTA, and WMTA mixed with distilled water is longer than that reported in literature [25, 26]; this variation could be related to the difference in cement's brands/compositions, techniques/needles, and standard conditions used for testing.

Using liquid “H” or “P” instead of water significantly (*p* < 0.05) reduced the setting time for all tested cement types. With PC and GMTA cement, the final setting time was reduced by two orders of magnitude; with WMTA, however, it was reduced by one order of magnitude when compared with those mixed with water. The final setting time recorded for PC and GMTA after being mixed with either liquid “H” or “P” is ~4 min. The final setting time recorded for WMTA after being mixed with liquid “H” or “P” is 22 and 63 min, respectively. The very short setting time (~4 min) is desirable for several applications, for example, root-end filling, perforation site repairs, and direct pulp capping procedures. The long setting time, however, could be useful for apical barrier application. As highlighted above, the reduction in setting time could be attributed to the accelerating action of calcium chloride [27]. Calcium chloride can easily penetrate into the cement's pores facilitating the hydration reaction and fasten the crystallization process [25]. Furthermore, when the liquid “P” is mixed with MTA cement, the high pH of the mix, confirmed by pH measurement, would increase the dissolution rate of the soluble calcium phosphate compound present in this liquid and subsequently fastens the hydration reaction. With liquid “H,” however, finely divided hydroxyapatite crystals, which present as one of its components, may act as nuclei for crystallization and hence hardening of the cement [28]. In both cases, a reduction in setting time was expected. According to the results of this study, the null hypothesis for the setting time is rejected.

Comparing the effect of both liquids “H” and “P,” there was no significant difference in the mean final setting time of cement mixed with H or P liquid except WMTA-H that has significantly (*p* < 0.05) shorter setting time than WMTA-P.

### 3.3. Degradation Study

**Figure 2:**
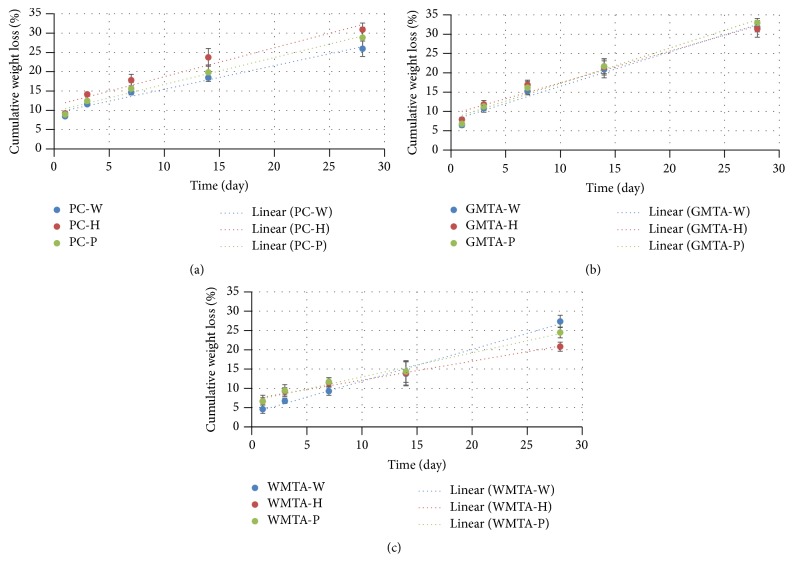
Cumulative weight loss (%) against time (day) for PC (a), GMTA (b), and WMTA (c).

The mean cumulative weight loss (%) for each cement at different time points is presented in Figure 2. At 24 h, the cumulative weight loss (%) ranged from 8.4 to 9.2% for PC group, 6.5 to 7.9% for GMTA group, and 4.6 to 6.6% for WMTA group. The cumulative weight loss (%) increased linearly with time for all tested cement types.

The degradation rate, calculated from the slope of the linear plot of cumulative weight loss (%) against time, indicated that using liquid “H” or “P” significantly reduced (*p* < 0.05) the degradation rate of WMTA when compared with their counterpart mixed with water. This reduction was prominent with “H” liquid compared to “P” liquid. Liquid “H” also produced a significant reduction in degradation of GMTA, but liquid “P” produced no significant difference from water. The degradation rate, however, was increased with the use of liquid “H” in particular or “P” with PC.

The reduction in degradation rate of both GMTA and WMTA could be attributed to the binding action of fumed silica as well as the reduction in the relative amount of bismuth oxide, associated with the addition of each liquid's components with respect to the powder weight. Bismuth oxide may interfere with the hydration reaction of MTA and remained as unbinding filler to the hydrated calcium silicate matrix [29, 30] producing more porous and weak cement [31]. Since PC has no bismuth oxide, the binding action of fumed silica alone could not be strong enough to reduce the degradation rate of PC. Furthermore, the liquid “H” has a stronger effect than “P” on reducing the degradation rate; this can be related to the presence of hydroxyapatite in “H” instead of calcium phosphate in “P” liquid. The dissolution of hydroxyapatite is lower than that of calcium phosphate and this could account for the stronger action of “H.” According to the results of this study, the null hypothesis for the degradation study is rejected.

### 3.4. X-Ray Diffraction Analysis

**Figure 3:**
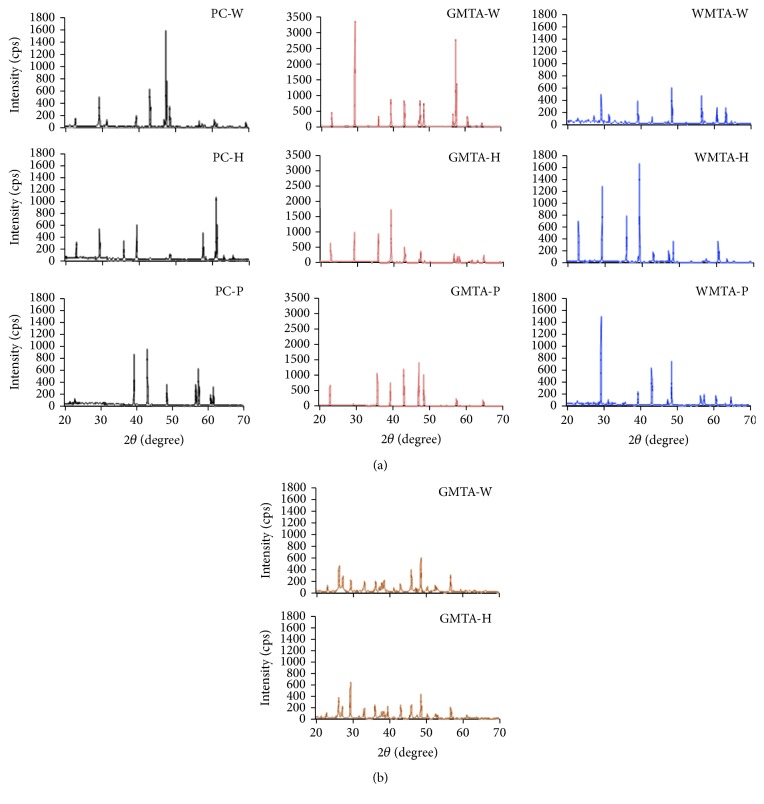
XRD patterns of precipitates collected from the surface of specimens of PC, GMTA, and WMTA cement groups (a) as well as from the bottom of the bottle used for the degradation study of GMTA-W and GMTA-H (b).

The precipitates seen on the surface of all tested cement types composed mainly of calcium carbonate “calcite.” Additional calcium hydroxide peak was identified from the precipitate of GMTA-H cement, Figure 3(a). Looking at the composition of the precipitates collected from the bottles used for the degradation study of GMTA-W and GMTA-H, calcite was also identified as the main phases for both samples. Additional hydroxyapatite peak was seen for GMTA-H precipitates, Figure 3(b). As is known, calcium hydroxide has been considered as the reaction product of these cement types; it may eventually interact with the phosphate ions forming amorphous calcium phosphate and finally hydroxyapatite [32].

### 3.5. Compressive Strength

**Figure 4:**
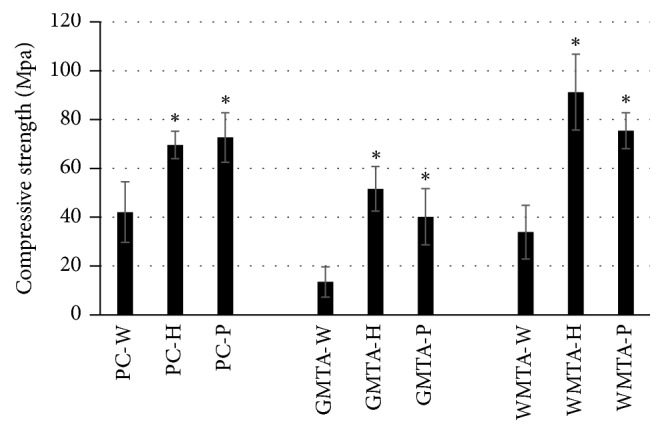
Mean compressive strength values for different cement used in this study. *∗* indicates significant difference from other groups of the same cement type (*p* < 0.05).

Using either “H” or “P” liquid instead of water significantly (*p* < 0.05) increased the compressive strength, Figure 4. Generally, both liquids significantly increased the 24 h compressive strength of all three cement types. Liquids “H” and “P” increased the compressive strength by 65% and 72% for PC, 280% and 197% for GMTA, and 170% and 122% for WMTA, respectively. This increase could be attributed to the presence of calcium phosphate or hydroxyapatite, in particular, which help in the hardening of the formed cement and hence in the strength development of the cement [23]. Furthermore, with calcium chloride, the formation of tetracalcium aluminum hydrate (C_4_AH_13_), which could be responsible for the high strength, rather than hydrogarnet (C_3_AH_6_) is more expected [25]. The increase in the compressive strength of both GMTA and WMTA mixed with either “H” or “P” liquid can be also attributed to the reduction of the weight% of bismuth oxide associated with the addition of the components of both experimental liquids with respect to the powder weight. During the hydration of MTA, the presence of more bismuth oxide resulted in the formation of calcium silicate bismuth hydrate (i.e., more porous cement [29, 30] with low compressive strength [31]). This increase in compressive strength is advantageous during perforation repair and pulp capping. According to the results of this study, the null hypothesis for the compressive strength test is rejected.

Within the same cement group, there was no significant difference in the compressive strength observed between those samples prepared with either “H” or “P” liquid. For example, the compressive strength of GMT-H (52 ± 9 MPa) is not statistically significantly different from that of GMT-P (40 ± 12).

### 3.6. Scanning Electron Microscopy

**Figure 5:**
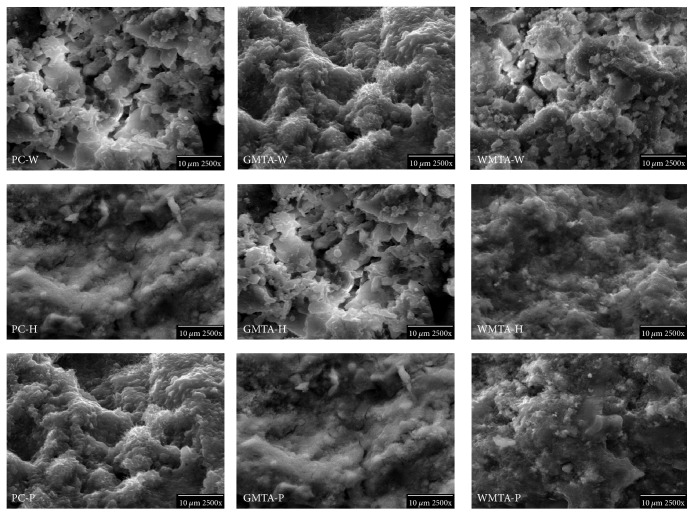
SEM micrographs of internal surfaces of PC, GMTA, and WMTA mixed with distilled water (coded as PC-W, GMTA-W, and WMTA-W, resp.), formulation “H” (coded as PC-H, GMTA-H, and WMTA-H, resp.), and formulation “P” (coded as PC-P, GMTA-P and WMTA-P, resp.). All samples mixed with distilled water showed porous and loosely packed structure compared to their counterparts mixed with “H” or “P” liquid.

As shown in Figure 5, all control cement types (PC-W, WMTA-W, and WMTA-W) showed porous and loosely packed structures compared to their experimental counterparts which had dense and solid-like structures. This dense structure could be also attributed to the presence of fumed silica and the subsequent formation of more calcium silicate hydrate binder [21, 22] that joins all components together and hence reduces the porosity. PC-H had an amorphous structure while the PC-P had needle-like and angular crystalline morphology. Furthermore, WMTA-H and WMTA-P showed more continuous homogenous structure than WMTA-W that had small needle-like structures growing in between large particles. The presence of needle-like crystals filling the spaces between the angular crystals could also account for the formation of more dense structure when liquid “H” or “P” was used for mixing. The formation of a highly consolidated and dense structure could also explain the significant increase in the compressive strength of cement mixed with liquid “H” or “P.”

## 4. Conclusions

From the findings of the present study, the use of experimental liquid “H” or “P” resulted in the following:

- No adverse effect on the high alkalinity of MTA
- A significant reduction by 1-2 orders of magnitude in the final setting time of tested cement
- A significant reduction in degradation rate of both GMTA and WMTA, but an increase with PC. Calcite was identified as the main phase precipitated during the degradation process. Additional calcium hydroxide or hydroxyapatite was seen on the surface or leached out from GMTA-H, respectively.
- A significant increase of the compressive strength (by 65% and 72% for PC, 280% and 197% for GMTA, and 170% and 122% for WMTA, resp.).
- A highly consolidated and dense cement.

## Figures and Tables

**Table 1:** Materials used in this study, liquids used for mixing, cements' codes and pH measurements.

Materials (*code*, manufacturer)	Liquids for mixing	Code after mixing with liquid		Immediate pH	30 min pH
*Portland cement type I* (*PC*, Quikrete® Number 1124, Atlanta, GA, USA)	(a) *Distilled water “W,”* provided by manufacturers. (b) *Experimental liquid “H.”* 0.3 g calcium chloride dihydrate (CaCl_2_·2H_2_O) + 0.075 g fumed silica (Aerosil EG 50, Evonik) + 0.3 g hydroxyapatite [Ca_10_(PO_4_)_6_(OH)_2_] + 1 ml distilled water. (c) *Experimental liquid “P,”* similar to “H” except that the 0.3 g hydroxyapatite was replaced by 0.3 g calcium hydrogen phosphate.	Powder mixed with distilled water	PC-W	12.20 ± 0.08	12.30 ± 0.20
		Powder mixed with liquid “H”	PC-H	11.90 ± 0.12	12.30 ± 0.05
		Powder mixed with liquid “P”	PC-P	11.97 ± 0.09	12.20 ± 0.02

*Grey ProRoot® MTA* (*GMTA*, Dentsply, Tulsa Dental Specialties, UK)	(a) *Distilled water “W.”* Provided by manufacturers. (b) *Experimental liquid “H,”* 0.3 g calcium chloride dihydrate (CaCl_2_·2H_2_O) + 0.075 g fumed silica (Aerosil EG 50, Evonik) + 0.3 g hydroxyapatite [Ca_10_(PO_4_)_6_(OH)_2_] + 1 ml distilled water. (c) *Experimental liquid “P.”* Similar to “H” except that the 0.3 g hydroxyapatite was replaced by 0.3 g calcium hydrogen phosphate.	Powder mixed with distilled water	GMTA-W	12.40 ± 0.01	12.60 ± 0.30
		Powder mixed with liquid “H”	GMTA-H	12.40 ± 0.10	12.70 ± 0.05
		Powder mixed with liquid “P”	GMTA-P	12.40 ± 0.07	12.60 ± 0.09

*White ProRoot® MTA* (*WMTA*, Dentsply, Tulsa Dental Specialties, UK)	(a) *Distilled water “W.”* Provided by manufacturers. (b) *Experimental liquid “H.”* 0.3 g calcium chloride dihydrate (CaCl_2_·2H_2_O) + 0.075 g fumed silica (Aerosil EG 50, Evonik) + 0.3 g hydroxyapatite [Ca_10_(PO_4_)_6_(OH)_2_] + 1 ml distilled water. (c) *Experimental liquid “P.”* Similar to “H” except that the 0.3 g hydroxyapatite was replaced by 0.3 g calcium hydrogen phosphate.	Powder mixed with distilled water	WMTA-W	12.10 ± 0.06	12.20 ± 0.20
		Powder mixed with liquid “H”	WMTA-H	11.60 ± 0.10	12.20 ± 0.10
		Powder mixed with liquid “P”	WMTA-P	11.90 ± 0.40	12.20 ± 0.10
